# An Unusual Case of Renal-Limited Giant Cell Arteritis

**DOI:** 10.7759/cureus.112089

**Published:** 2026-07-05

**Authors:** Sarah Crowder, Prajwol Pant, Mario Madruga, Steve Carlan

**Affiliations:** 1 Internal Medicine, Orlando Regional Medical Center, Orlando, USA; 2 Nephrology, Blue Ridge Nephrology Associates, Christiansburg, USA; 3 Obstetrics, Orlando Regional Medical Center, Orlando, USA

**Keywords:** elevated bun hemodialysis, end-stage renal disease (esrd), fatal outcome, giant cell arteritis (gca), renal arteritis

## Abstract

It is widely accepted that giant cell arteritis (GCA) is a disease of the carotid branches. There are very few cases describing disseminated disease or visceral involvement. Isolated renal GCA presenting with renal failure is even more unusual.

A 76-year-old male with no known medical history presented to the emergency department with three weeks of weakness, swelling, oliguria, and shortness of breath. He was started on hemodialysis for renal failure. After microscopic urinalysis revealed red blood cell casts, a kidney biopsy was performed and showed granulomatous arteritis suggestive of GCA in the renal vasculature. Despite an aggressive corticosteroid regimen, the patient remained dependent on hemodialysis until his death from an unrelated cause.

The leading diagnosis throughout most of the workup in this case was end-stage renal disease in the setting of previously undiagnosed chronic kidney disease. A thorough workup revealed a far less common disease process, which significantly altered the patient’s treatment course and highlighted the importance of maintaining a broad differential.

## Introduction

Giant cell arteritis (GCA) has a predilection for the intra- and extracranial carotid branches [1] and the aorta [2]. Classically, symptoms of GCA include headache, visual defects including amaurosis fugax or even permanent vision loss [1], jaw claudication, temporal tenderness to light touch [3], and constitutional symptoms such as fever and malaise [4]. The diagnosis is confirmed histologically, usually by temporal artery biopsy [5]. It is labeled a large-vessel disease because the immune system preferentially targets arteries rich in elastic tissue [5].

GCA rarely results in renal failure; one study comparing 68 patients with GCA found that none had decreased renal function at the time of diagnosis [6]. Rarely, renal involvement can be part of broader systemic disease, as in disseminated visceral GCA [7]. When renal damage occurs, it is often prerenal due to involvement of the renal arteries. Granulomatous lesions occlude the lumen of the vessel, leading to reduced blood flow, thereby reducing the filtration mechanism of the kidney or even causing an infarct [8]. However, a few scattered cases of GCA limited to the small vessels of the renal vasculature have been documented. In these cases, kidney function was disturbed in an intrarenal pattern, with defacement of the capillaries that interface with the glomeruli. These cases fall under the category of granulomatous interstitial nephritis (GIN). These patients presented with signs of kidney damage, including hypertension [9] and elevated creatinine [10,11], as well as signs of intrinsic kidney damage, including hematuria [10,12], red cell casts [11], and proteinuria [12]. Evidence of kidney damage often presents in the context of constitutional symptoms such as fever [11,12], malaise [10,12], and myalgia [11,12]. All reviewed cases had significantly elevated erythrocyte sedimentation rates (ESRs) [10-12], ranging from 69 [11] to 120 [10]. The diagnosis was established by clinical correlation with histologic findings of granulomatous arteritis within the renal vasculature [10-12]. Renal GCA is extremely difficult to diagnose without a renal biopsy because it often lacks the typical head and neck symptoms associated with classical GCA. Renal GCA differs from other common causes of renal failure through its distinct pathophysiology, histology, and urinalysis profile [9-12]. In previous cases treated with high-dose corticosteroids, the prognosis was variable and included improvement in renal function [10-12] and progression to classic symptoms of GCA with temporal artery involvement [12].

In cases of GIN, other granulomatous diseases must be excluded, especially since the symptoms can be vague and variable. The most common causes of GIN vary by region and applicable risk factors [13]. Disease processes to be considered include pauci-immune vasculitis, autoimmune disease such as systemic lupus erythematosus and scleroderma, infectious causes such as tuberculosis, systemic inflammatory syndromes such as sarcoidosis, and drug-induced granuloma formation. Diagnostic tools include a detailed history of risk factors and underlying medical conditions, serologic studies to point to infection and autoimmune processes, and histologic findings [9,13].

## Case presentation

A 76-year-old Caucasian male with an unknown medical history presented to the emergency department with three weeks of progressive weakness, swelling, shortness of breath, and decreased urination. He had not seen a doctor for 20 years and took no prescription medications. He denied cough, fever, muscle aches, and gastrointestinal symptoms. There were no noted visual, neurologic, or vascular symptoms or disturbances. He was normotensive and afebrile. Initial laboratory studies showed a creatinine of 24.5 mg/dL (0.6-1.1 mg/dL), a blood urea nitrogen (BUN) of 246 mg/dL (7-20 mg/dL), and hyperkalemia with peaked T waves on ECG. Chest X-ray was negative for cardiomegaly but showed a tortuous aorta. Transthoracic echocardiogram and liver function studies ruled out cardiac and hepatic causes of his clinical volume overload. Nephrology consultation was obtained, and he was started on hemodialysis three times weekly and experienced improvement in symptoms.

Further workup was performed to explore potential causes of the patient’s renal failure. The patient was found to have normocytic anemia with a hemoglobin of 8.7 g/dL (13.5-18.0 g/dL) and a parathyroid hormone (PTH) level of 240 pg/mL (0-65 pg/mL). Further evaluation of the anemia did not reveal any additional pathologies. Microscopic urinalysis was positive for red blood cells and red cell casts, prompting further diagnostics. A renal ultrasound ruled out hydronephrosis. The glycated hemoglobin (HbA1c) level was 5.7%. Levels of C3, C4, antinuclear antibody (ANA), cytoplasmic antineutrophil cytoplasmic antibody (c-ANCA), perinuclear antineutrophil cytoplasmic antibody (p-ANCA), serum protein electrophoresis (SPEP), urine protein electrophoresis (UPEP), serum free light chains, and cryoglobulin were all measured and found to be noncontributory. The ESR was significantly elevated at 108 mm/h (0-15 mm/hour) (Table 1).

**Table 1:** Laboratory variables. ECG: electrocardiogram; ANA: antinuclear antibody; c-ANCA: cytoplasmic antineutrophil cytoplasmic antibody; p-ANCA: perinuclear antineutrophil cytoplasmic antibody; SPEP: serum protein electrophoresis; UPEP: urine protein electrophoresis

Test	Result	Reference range	Interpretation
Creatinine	24.5 mg/dL	0.7-1.3 mg/dL	Markedly elevated
Blood urea nitrogen (BUN)	246 mg/dL	7-20 mg/dL	Markedly elevated
Potassium	Elevated (peaked T waves on ECG)	3.5-5.0 mmol/L	Hyperkalemia
Hemoglobin	8.7 g/dL	13.5-17.5 g/dL	Low; normocytic anemia
Parathyroid hormone (PTH)	240 pg/mL	15-65 pg/mL	Elevated
Hemoglobin A1c	5.70%	<5.7%	Normal
Erythrocyte sedimentation rate (ESR)	108 mm/h	0-22 mm/h	Markedly elevated
Microscopic urinalysis	Red blood cells and red cell casts present	None	Active urinary sediment
C3, C4, ANA, c-ANCA, p-ANCA, SPEP, UPEP, serum free light chains, cryoglobulin	Negative/within normal limits	-	Noncontributory

**Figure 1:**
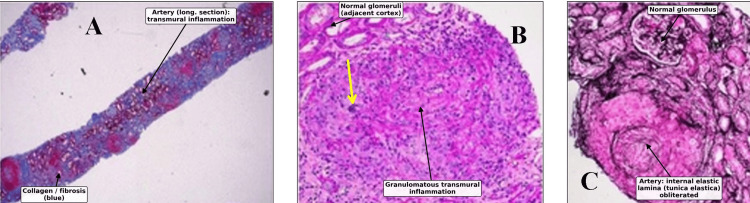
Renal biopsy demonstrating granulomatous arteritis consistent with renal-limited giant cell arteritis (GCA). (A) Masson trichrome, low power: a longitudinally sectioned interlobular artery showing transmural inflammation with mural and interstitial collagen deposition (blue), consistent with a chronic fibrosing arteritis. (B) Hematoxylin and eosin: transmural granulomatous inflammation of the artery, with adjacent renal cortex containing histologically normal glomeruli; no crescents or fibrinoid necrosis are present. Multinucleated giant cell at the yellow arrow. (C) Elastic (Verhoeff-Van Gieson) stain: obliteration of the internal elastic lamina (tunica elastica) by the inflammatory infiltrate with a preserved normal glomerulus, a hallmark of GCA, but not entirely specific for GCA, and may overlap other forms of granulomatous vasculitis.

A kidney biopsy was obtained. Pathology demonstrated granulomatous arteritis with mild-to-moderate interstitial fibrosis and limited glomerular sclerosis, as shown in Figure 1.

No crescents or necrotizing vasculitis were identified. A diagnosis of renal-limited GCA with secondary granulomatous arteritis was made.

The patient continued to deny headache, temporal pain, jaw claudication, or muscle aches. Further diagnostic testing to rule out involvement of the temporal arteries and aorta was offered, but the patient declined. The patient was presented with various treatment options, including high-dose steroids and more specialized treatments, and opted to try steroids through shared decision-making. He started on methylprednisolone 1g IV for three days, followed by a prolonged oral prednisone taper beginning with 60mg. His treatment regimen was based on the recommended steroid course for GCA-induced loss of vision because both clinical manifestations suggest microvascular damage. Despite patient-reported compliance with therapy, the patient failed to regain kidney function and remained dependent on hemodialysis until his death from unrelated causes a year later.

## Discussion

Our case began with identifying and ruling out more likely causes of both renal failure and vasculitis. Initially, the favored differential was previously undiagnosed chronic kidney disease progressing to end-stage renal failure, which was supported by the lack of available medical history, the patient’s advanced age, and elevated PTH levels. However, urinalysis clearly demonstrated red cell casts, indicating an underlying glomerulonephritis, and so a kidney biopsy was pursued. Once granulomatous vasculitis was identified, negative serologic studies decreased the likelihood of pauci-immune vasculitis and autoimmune disease. The patient denied chronic use of any medications, excluding drug-induced causes. His history lacked risk factors for tuberculosis, and he had a chest X-ray that was negative for lung pathology, so tuberculosis was deemed unlikely. Finally, histologic findings specific to GCA, including obliteration of the elastic lamina, steered the diagnosis towards renal-limited GCA rather than systemic granulomatous diseases, such as sarcoidosis, which lack such changes.

Our case was notable for the absence of constitutional symptoms upon the patient’s arrival at the ED, in contrast to previously reported cases [9-11]. Furthermore, the patient never displayed symptomatic disease progression to temporal arteries or disseminated visceral GCA, although involvement of the temporal arteries is impossible to exclude given the patient’s refusal of a temporal artery biopsy. This case underscores the importance of maintaining a broad differential in patients with renal failure. We propose that microscopic urinalysis should be obtained regardless of whether institutional policy mandates microscopic analysis of every urine specimen in order to rule out glomerulonephritis in cases of acute renal failure. While uncommon, this case underscores the importance of keeping vasculitis on the differential, even in less commonly affected organ systems, especially when the ESR is elevated.

Given the prevention of feared sequelae of GCA, such as vision loss, with prompt steroid use [4], we extrapolated that high-dose corticosteroids might improve kidney function. Despite steroid treatment, our patient never recovered kidney function and remained dependent on hemodialysis until his death approximately one year later. While there are isolated cases of resolution of kidney failure secondary to GCA in the literature following steroid therapy [9-11], this may be explained by those patients presenting earlier in the progression of the disease course; for instance, a woman successfully treated with steroids was not yet oliguric [9], whereas our patient was anuric. The failure of therapy in this case is consistent with the disease prognosis in other manifestations of an advanced course of GCA. For instance, it is widely accepted that prompt treatment is critical for vision loss associated with GCA, because once amaurosis fugax progresses into longer-lasting vision loss, it is often permanent. This case underscores the importance of prompt management of GCA when end-organ failure is threatened.

The diagnosis can be difficult without a renal biopsy. The differential diagnosis of granulomatous renal arteritis or nephritic sediment should be considered and excluded, including ANCA-negative vasculitis, polyarteritis nodosa, anti-glomerular basement membrane (anti-GBM) disease, infection-associated vasculitis, cholesterol embolization, IgA nephropathy or other immune-complex disease, and drug-related or systemic inflammatory disease [14]. GCA is known to overlap with other granulomatous vasculitides, such as Takayasu arteritis (TAK) and granulomatosis with polyangiitis (GPA). This overlap reflects shared CD4+ T cell-mediated granulomatous inflammation that can destroy the arterial wall [15]. However, GCA is unique in classic cranial artery involvement, with an onset almost exclusively in patients over 50 and distinct genetic backgrounds [15]. In this case, GCA was favored in part because of the patient’s age and the absence of limb claudication. The fact that aortic or branch-vessel imaging was not obtained because the patient declined further testing is a limitation in this case report.

Urine protein quantification with a protein/creatinine ratio or 24-hour protein excretion and dialysis parameters to further characterize the renal presentation are helpful elements in diagnosis and management [16], but unfortunately, in this case, those were not available.

One of the limitations of the case is that renal-limited GCA is a plausible diagnosis; the absence of temporal artery biopsy or vascular imaging limits definitive exclusion of occult systemic large-vessel disease.

## Conclusions

This case underscores the importance of maintaining a broad differential, even in patients with seemingly routine complaints. Throughout most of the workup, the leading diagnosis was end-stage renal disease in the setting of previously undiagnosed chronic kidney disease, given the patient’s lack of prior medical care. A careful workup revealed a far less common disease process, which significantly altered the patient’s treatment course.

Since the patient declined temporal artery biopsy, aortic imaging, and additional large-vessel assessments, it is not possible to definitively diagnose "renal-limited GCA.” The diagnosis was primarily based on renal biopsy results and clinical exclusion, rather than a full assessment of cranial or systemic large-vessel involvement. Renal-limited GCA was the most likely diagnosis, highlighting the importance of considering vasculitis in cases of renal failure that present with active urinary sediment and significantly elevated inflammatory markers.
